# Heat induces end to end repetitive association in *P. furiosus*l-asparaginase which enables its thermophilic property

**DOI:** 10.1038/s41598-020-78877-z

**Published:** 2020-12-10

**Authors:** Pankaj Sharma, Rachana Tomar, Shivpratap Singh Yadav, Maulik D. Badmalia, Samir Kumar Nath, Ashish, Bishwajit Kundu

**Affiliations:** 1 CSIR-Institute of Microbial Technology, Sec 39 A, Chandigarh, 160036 India; 2 Kusuma School of Biological Sciences, Indian Institute of Technology Delhi, New Delhi, 110016 India

**Keywords:** Biophysics, Structural biology

## Abstract

It remains undeciphered how thermophilic enzymes display enhanced stability at elevated temperatures. Taking l-asparaginase from *P. furiosus* (PfA) as an example, we combined scattering shapes deduced from small-angle X-ray scattering (SAXS) data at increased temperatures with symmetry mates from crystallographic structures to find that heating caused end-to-end association. The small contact point of self-binding appeared to be enabled by a terminal short β-strand in N-terminal domain, Leu^179^-Val-Val-Asn^182^ (LVVN). Interestingly, deletion of this strand led to a defunct enzyme, whereas suplementation of the peptide LVVN to the defunct enzyme restored structural frameworkwith mesophile-type functionality. Crystal structure of the peptide-bound defunct enzyme showed that one peptide ispresent in the same coordinates as in original enzyme, explaining gain-of lost function. A second peptide was seen bound to the protein at a different location suggesting its possible role in substrate-free molecular-association. Overall, we show that the heating induced self-assembly of native shapes of PfA led to an apparent super-stable assembly.

## Introduction

Intrinsic resistance to temperature induced denaturation and subsequent loss of functionality in hyperthermophilic proteins remains a working puzzle for biophysical chemists. Prior studies have searched for correlations between sequence or structural features with the presence or absence of strong ion-pairing networks, salt-bridge like interactions, conformational rigidity, compact packing, shortening or deletion of surface loops, hydrophobic core, dimerization/buried surfaces, subunit association, disulfide formation etc*.*^1–4^. In thermophilic proteins, surface residues are usually involved in salt bridges and ion-pair networks while the core is prevalent with hydrophobic residues containing branched apolar amino acids^2,3^. Such composition results in an optimized structure that can resist an external stress factors including temperature. Furthermore, the literature suggests that thermophilic proteins have the ability to exist in a wider range of conformations or assembled states and this additional entropy dampens the heat-induced deformities or misfolding^5^. One example from the hyper-thermophilic archaea, *Methanococcus janaschii* Hsp 16.5 has been shown to have a dynamic quaternary structure where its subunits freely and reversibly exchange with each other at high temperature conditions^5^. Furthermore, the temperature induced functional associations of protein molecules have been suggested as a way by which thermophilic proteins evade the stress^6–8^. The formation of such heat-induced higher order oligomeric species has been shown through native PAGE for the hyperthermophilic proteins, Fe-Superoxide Dismutase (tcSOD)^9^ as well as sulfurtransferase from bacterium *Aquifex aeolicus*^10^. Interfacial mutational studies led to decrement in native-like heat induced intermolecular associations, indirectly supporting oligomerization-dependent stabilization^11,12^. Along with inherent flexibility in the thermophilic heat shock proteins, the Nano-ESI-mass spectrometry confirmed the formation of oligomers at increased temperatures^13^. Another theory suggests the catalytic speed of enzyme reactions as an evolutionary driver for thermo-adaptation for sustenance under thermal stress^14^. Though these studies indicate that some inherent stabilization or oligomerization features exist in thermophilic proteins, a discrete set of information that may aid researchers in designing or tinkering activity of thermophilic proteins still elude us.

The prime reason for this uncertainty is that most of the structural information on thermophilic proteins is obtained from low temperature studies which many times miss out on information on their primary nature. In other words, relevant shape-function information cannot be extrapolated from partial low temperature data. Variable temperature solution light scattering and/or SAXS experiments have provided important findings about influence of temperature on protein shape or association levels which expand our understanding^15–18^. Previously, we provided a detailed structural and mechanistic insight of thermophilic l-asparaginase from *Pyrococcus furiosus* in its full-length (PfA), and a conjoined functional version (cPfA) (domain architecture shown in Supplementary Fig. S1)^19,20^. SAXS and crystal diffraction data analysis brought forth that despite lacking a 19-residue long linker which connects the two domains, namely the N- and C-halves (N-PfA and C-PfA, respectively), cPfA adopts a solution shape and crystalline state that is comparable to wild type full-length protein PfA with improved specific-activity^19^. Both, PfA and cPfA showed comparable thermophilic profile^19^, and thus offered a good case study to understand shape-function correlation. The structural studies on these thermophilic proteins were done at low temperatures which were thus limiting in understanding their stability and activity at elevated temperatures^19,20^. As presented in the current study, the high temperature SAXS data along with enzyme assay allowed us to obtain new information on the structural organization of PfA and cPfA responsible for providing stability and facilitating these proteins to remain active at higher temperatures. Here we show that rationalized deletion of a small segment involved in heat-induced association led to defunct protein (dcPfA). Supplementation of synthetic peptide corresponding to this segment to dcPfA reinstated structural association and rescued the enzyme from activity loss, confirming the role of this stretch in the thermophilic function of the enzyme. Structure–function approach followed in the present study can work as a template to understand other systems.

## Results

### Variable temperature SAXS data on PfA and cPfA

**Figure 1:**
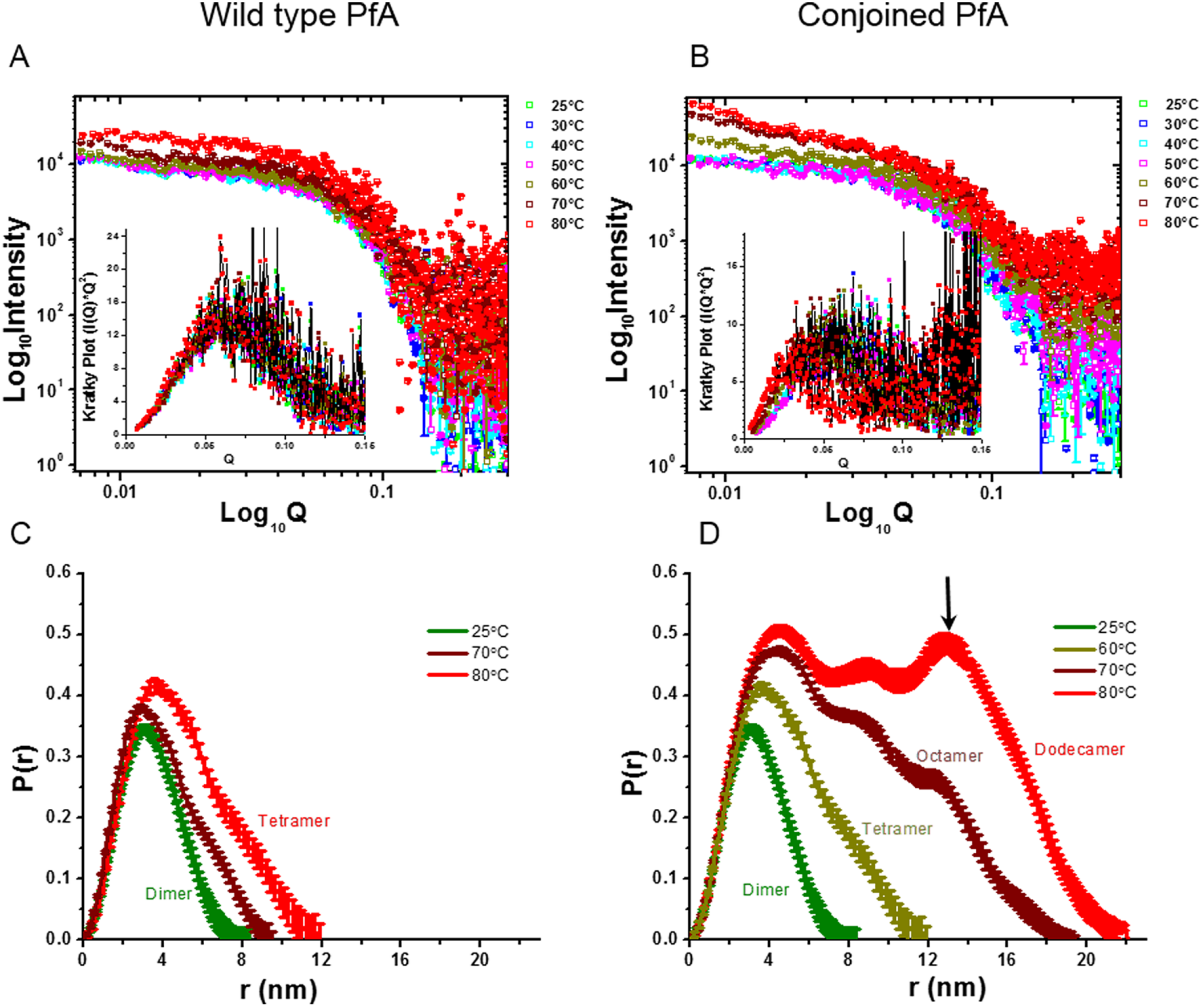
Variable temperature SAXS data collected on wild type (PfA) and conjoined PfA (cPfA). (**A**) SAXS I(Q) profiles collected on wild type PfA at 6 mg/mL from 25 to 80 °C are presented in double logarithm format. Inset shows the Kratky plots of the same datasets. (**B**) SAXS datasets acquired from conjoined PfA at 6 mg/mL at different temperatures are presented in double log format. Inset shows the Kratky plots of the same. Computed P(r) profiles from the representative datasets at different temperatures (color matched) from wild type (**C**) and conjoined (**D**) PfA are shown here (dimensions of r is in nm). Predominant association state as estimated from I_0_ values from Guinier analysis of the SAXS I(Q) profiles are mentioned next to the computed curve.

To gain insight into the shape and association of PfA and cPfA in solution, we acquired SAXS data at different temperatures ranging from 25 to 80 °C (Supplementary Table S1). Plots of Log_10_Intensity vs. Log_10_Q affirmed that there was no aggregation in the samples of PfA and cPfA at any temperature (Fig. 1A,B). Though the scattering intensity profiles increased to higher values from 60 to 80 °C for both the proteins, the change was more significant for cPfA protein. Kratky analysis of all the datasets showed bell shaped profiles confirming that both the proteins were globular in solution at all temperatures (Fig. 1A,B, insets). In particular, all the Kratky plots for the datasets clustered at similar Q values supporting similarity in the particle size of PfA at all temperatures. In contrast, at increased temperatures, the peak in Kratky plots for cPfA shifted towards lower Q values indicating increase in particle size. Similar shifts were previously reported and validated for heat-induced native-like association of lysozyme^15^.

Having confirmed the globular nature of these protein molecules, Guinier analysis for globular particles provided a reliable estimation of R_g_ values as well as estimated intensity at zero angles (I_0_) at different temperatures (Table 1). It was clear that heating induced an increase in the average R_g_ values of the protein molecules of PfA and cPfA in solution. The values were close to 2.5 nm for PfA and cPfA from 25 to 60 °C and 25–50 °C, respectively. R_g_ values for PfA increased to 3.12 and 3.77 nm at temperatures 70 °C and 80 °C, respectively. In contrast, the size of cPfA molecules increased to 3.75, 5.98 and 7.56 nm at 60 °C, 70 °C and 80 °C, respectively suggesting a significant increase in particle size. It is pertinent to mention here that the SAXS datasets were also collected for both proteins at around 3.2 and 8.5 mg/mL, and data analysis provided similar shape parameters, indicating that datasets collected at 6 mg/mL were a good representation of the shape induced changes in the proteins. Extrapolated I_0_ values from Guinier analysis are tabulated in Table 1. In comparison to the standard used, the molecular masses of predominant (or average) scattering species in sample at that temperature were calculated. Analysis revealed that PfA remains predominantly dimer (about 70 kDa) from 25 to 50 °C. Upon further increase in temperature, the molecular masses of particles in solution were clearly bigger than dimer, and at 80 °C the estimated mass was closer to that of tetramer of PfA. Similarly, cPfA appeared to adopt a dimeric status from 25 to 50 °C, but further heating induced the protein to associate as tetramer, octamer and dodecamer (Table 1).

**Table 1:** The radius of gyration (*R*_*G*_) and molecular mass (*kDa*) calculated for PfA and cPfA from forward scattering intensities (*I*_0_) estimated from the Guinier analysis of the SAXS data are tabulated below.

Temperature (°C)	Guinier analysis			Scattering shape from Guinier plot for rod-shape (association state)
	*Rg *(nm)	Intensity (I0)	Calculated Mol. mass (kDa)	
Lysozyme*	1.40 ± 0.02	297	14.2	Globular (monomer)
**PfA (6 mg/mL)**				
25	2.42 ± 0.11	12,491	70.1	Globular (dimer)
30	2.50 ± 0.13	12,438	69.8	Globular (dimer)
40	2.49 ± 0.09	12,848	72.1	Globular (dimer)
50	2.52 ± 0.45	12,776	71.7	Globular (dimer)
60	2.43 ± 0.38	15,057	84.5	Globular (mixed order)
70	3.12 ± 0.3	19,691	110.5	Globular (mixed order)
80	3.77 ± 0.37	24,609	138.1	Globular (tetramer)
**cPfA (6 mg/mL)**				
25	2.43 ± 0.08	12,135	68.1	Globular (dimer)
30	2.49 ± 0.11	12,367	69.4	Globular (dimer)
40	2.52 ± 0.28	12,687	71.2	Globular (dimer)
50	2.49 ± 0.59	12,545	70.4	Globular (dimer)
60	3.75 ± 0.55	24,507	135	Globular (tetramer)
70	5.98 ± 0.60	48,648	273.1	Globular (octamer)
80	7.56 ± 0.68	67,360	378	Globular (dodecamer)

*Estimated for 1 mg/mL of Lysozyme for same exposure time.

*N.B*. the molecular mass was calculated using this formula: $\frac{{I_{l} }}{{m_{l} \times c_{l} \times t_{l} }} = \frac{{I_{g} }}{{m_{g} \times c_{g} \times t_{g} }}$ where, *I* = intensity at zero angles, *m* = molecular mass, *c* = concentration, and time t_l_ = t_g_.

Similar observations were made for samples at 3.2 and 8.5 mg/mL, but the cPfA samples indicated non-integral polydispersity in association at most temperatures. This was another reason to consider SAXS datasets for samples at 6 mg/mL as the intensity values upheld association levels close to integral values. These experiments indicated that though the dimer existed at ambient temperature, heating induced higher order association.

### SAXS data based shape models and their comparison with crystal structure based information

Previously, we reported the SAXS data based shape parameters and envelope models of PfA and cPfA, which confirmed that both the proteins dimerized at 10 °C (R_g_ ~ 2.7 nm)^19^. These were later supported by their respective crystal structures^19^. From 25 to 50 °C, the R_g_ of these proteins were close to 2.5 nm which correlated well with the calculated R_g_ of PfA crystal structure (PDB ID: 4Q0M)^19^. Also, the calculated χ^2^ values were in the range of 1.1–1.2 between the experimental SAXS data (Q range 0.07–0.25 nm^−1^) and the theoretical profile of the crystal structure of dimer, confirming that the proteins adopted dimeric status in solution in this temperature range. Before shape restoration of dimeric and the other associated versions, we auto-computed the distribution of pairwise interatomic vectors, P(r) present inside the SAXS intensity profiles of proteins (Fig. 1C,D). The profiles indicated that at 80 °C, the PfA adopted a shape characterized by D_max_ of 12 nm supported to be tetramer from I_0_ values (Fig. 1C; Table 1). In between, at 70 °C, the computed P(r) indicated that PfA adopts a mixture of populations of dimer and tetramer (Fig. 1C). As expected from I_0_ values, the cPfA also adopted a shape characterized by D_max_ of 12 nm for tetramer but at 60 °C. The P(r) profiles were characterized by D_max_ of ~ 20 and 22 nm for the estimated octamer and dodecamer species of cPfA at 70 °C and 80 °C, respectively (Fig. 1D). The P(r) profile solved for dodecamer indicated that the vector profiles increased again at around ~ 15 nm (Fig. 1D, shown by arrow). This may be suggestive of some sort of spiral shape where the extended molecular shape “comes back” to another part of the molecule.

**Figure 2:**
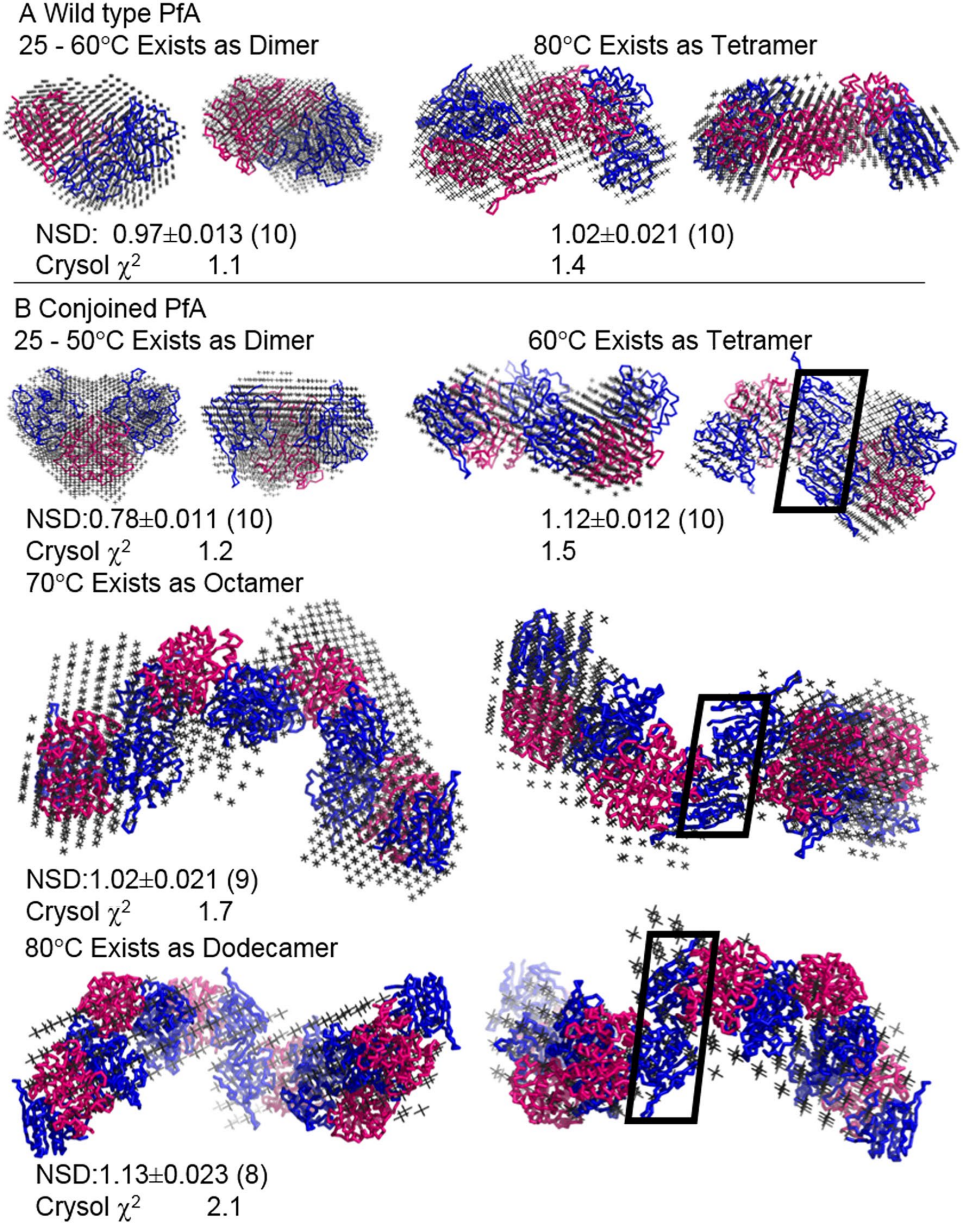
SAXS data based average of scattering shapes restored for the wild type (PfA) and conjoined PfA (cPfA) at different temperatures. (**A**) On the left is, the envelope shape of dimeric form of wild type PfA existing in solution from 25 to 60 °C. For comparison, inertial axis overlay with the dimer from PDB ID: 4Q0M has been shown^19^. The right panel shows the SAXS data based tetramer of wild type PfA present in solution at 80 °C. The dummy atom model has been overlaid on a symmetry based tetramer from the crystal structure of the same protein (PDB ID: 4Q0M). (**B**) Top left shows the dimeric shape of conjoined PfA which predominantly exists in solution from 25 to 50 °C. Crystal structure of the same protein (PDB ID: 5CBP) was used to overlay and compare the shape profiles. Top right panel shows the tetramer of conjoined PfA which agrees well with the computed tetramer from its crystal structure using symmetry mates. Middle and lower panels show the comparison of the SAXS data based shapes of octamer and dodecamer of conjoined PfA which are dominantly present in the solution at 70 and 80 °C, respectively. For automated overlay using inertial axes, same associations were generated from symmetry mates of crystal structure PDB ID: 5CBP. Below every model, normalized spatial disposition (NSD) between the ten models computed have been mentioned. Of the ten independent models calculated for each dataset, number of models which could be considered for averaging has been mentioned in the parenthesis. Finally, the comparison between the calculated SAXS profile of the averaged model presented here with the experimentally acquired SAXS profile have been mentioned below the respective figure as χ^2^ value.

Using the integrated plugins in ATSAS suite of programs, ten uniform density dummy residue models were solved for each shape, subsequently compared with crystal structures and the associations were perceived from their symmetry mates (selected using shape profile of solved SAXS envelope and parameters) (Fig. 2). All ten models were superimposed by aligning their inertial axes and those more than twice the deviation of calculated normalized spatial disposition (NSD) value were discarded from averaging. The NSD values are given for each SAXS based averaged model in Fig. 2 and the number of models averaged to obtain it are mentioned in parenthesis. All models of PfA and cPfA were compared with their PDB submissions, 4Q0M^19^ and 5CBP (crystal structure of cPfA at 37 °C, described ahead), respectively. CRYSOL program^21^ based computed χ^2^ values provided quantitative description of similarity between the crystal structure based models and SAXS based average envelope (values close to 1 are considered identical). Of various probabilities in considering association of tetramer to dodecamer, only one orientation satisfied the experimentally obtained profile from SAXS. A quick comparison revealed that a certain interaction of N-N′-terminal acted as a pivotal junction for heat induced progressive interaction.

### Effect of temperature on structure of PfA

**Figure 3:**
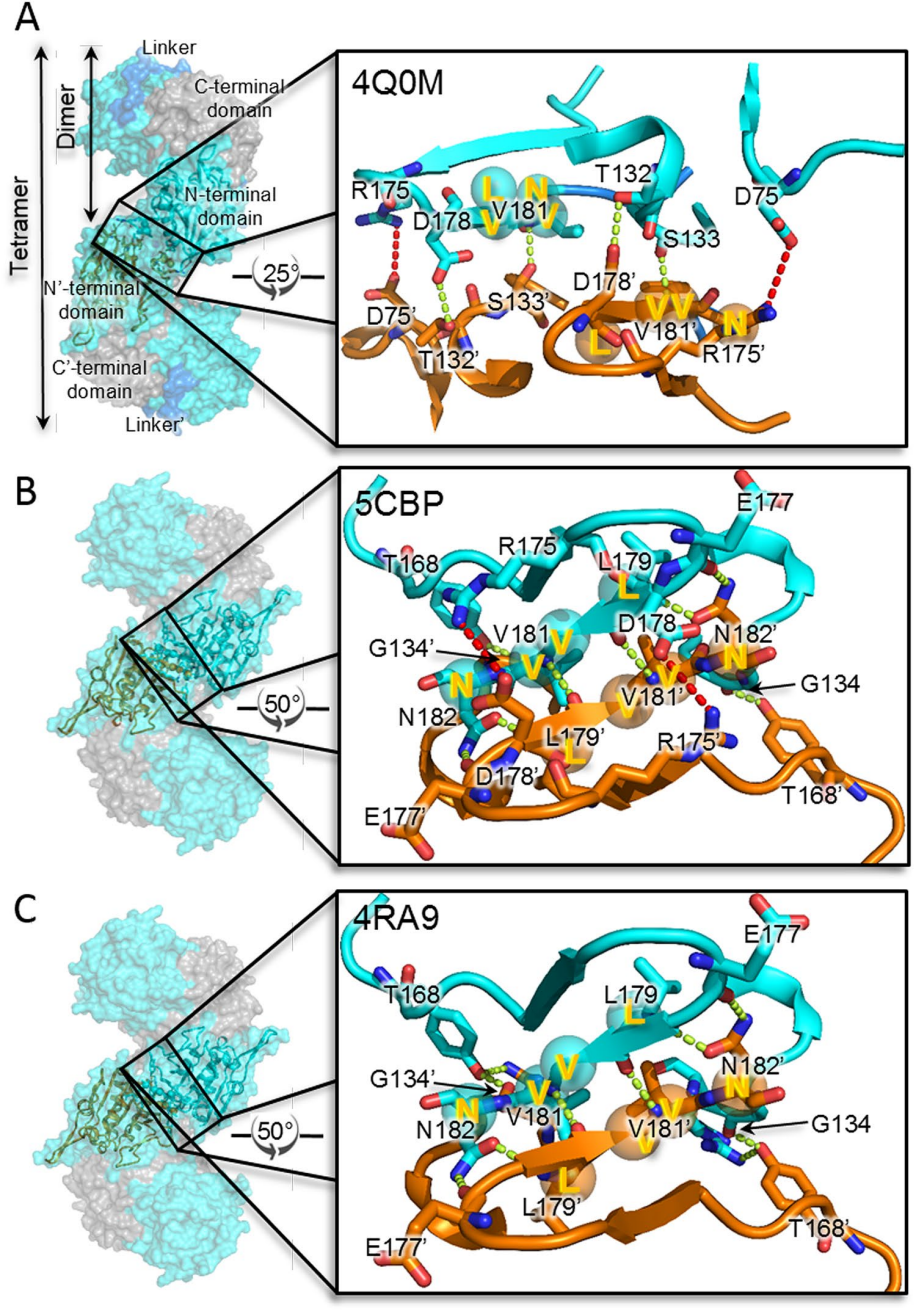
Interaction analysis of symmetry based tetramer from the crystal structures of different PfA variants. (**A**) Wild type PfA (PfA) crystals grown at 18 °C, (PDB ID: 4Q0M)^19^. (**B**) conjoined PfA (cPfA) crystals grown at 37 °C, (PDB ID: 5CBP) and (**C**) 18 °C, (PDB ID: 4RA9)^19^. Left panel shows surface representation (N-terminal domains, cyan and C-terminal domains, grey) of symmetry based tetramer structures. Right panel shows enlarged view of interacting residues of adjacent N-terminal domains. Interacting N-terminal domains of adjacent protein molecules are in cartoon representation marked as N (cyan) and N′ (orange) terminal domains.

Taking insights from SAXS experiments and the crystal structures previously reported by our group^19^, PDBsum^22^ based interaction analysis of N-terminal domains of symmetry-based PfA tetramer was carried out (PDB ID: 4Q0M)^19^. The results showed that there were 4 hydrogen bonds, 2 salt bridges and 44 hydrophobic interactions between these domains (Supplementary Table S2, Supplementary Fig. S2). Residues, T132 and S133 formed H-bonds with D178′ and V181′ and residues, D75 and R175 formed salt bridges in a reversible manner between these domains (Fig. 3A). Importantly, most of the residues involved in interactions belong to the terminal region of the N-terminal domain depicting that this region might be involved in molecular associations.

To visualize the structural changes accompanying PfA under high temperature conditions, crystallization was setup between 30 and 50 °C. However, no crystals were produced under these conditions. Since the residues involved in interactions (described above) were also present in the linker-less PfA variant cPfA, we attempted to crystallize the latter under high temperature conditions. While cPfA crystallized at 37 °C and diffracted to 2.36 Å resolution, the unit cell parameters of this crystal (PDB ID: 5CBP, Table 2) were identical to the previous crystal structure of cPfA where crystals were grown at 18 °C (PDB ID: 4RA9)^19^. Detailed analysis showed negligible structural differences between both the structures (R.M.S.D at Cα, 0.12 Å). Moreover, almost identical interactions were formed between symmetry related adjacent N-terminal domains of both structures (Fig. 3B,C).

**Table 2:** Crystallization table.

	cPfA at 37 °C	cPfA with L-V-V-N peptide	dPfA with L-V-V-N peptide
**Data collection**			
Resolution (Å)	50–2.36	50–2.04	50–2.61
Space group	P6522	P6522	P6522
Unique reflections	19,854	29,169	13,346
**Unit cell parameters**			
a (Å)	90.55	92.01	91.31
b (Å)	90.55	92.01	91.31
c (Å)	190.04	187.08	186.97
α	90°	90°	90°
β	90°	90°	90°
γ	120°	120°	120°
Completeness (%)	99.9 (99.9)	94.5 (82)	90.9 (88.8)
R merge	0.08 (0.76)	0.11 (0.64)	0.10 (0.45)
Redundancy	13.3 (9.9)	13.5 (13)	13.4 (13.7)
Average *I/σ (I)*	38.9 (2.3)	13.2 (3.4)	15.9 (5.7)
**Refinement**			
R_work_ (%)	19.8	21.28	20.54
R_free_ (%)	24.3	24.17	24.68
Solvent content	58.47	35.9	59.7
No. of chains	A,B	A,B,D	A,B,C,E
**R.M.S.D. from ideality**			
Bonds (Å)	0.008	0.007	0.009
Angles (°)	1.138	1.041	1.122
Wilson B-factor (Å^2^)	49.5	30.3	41.0
**Ramachandran plot: residues**			
Most favoured (%)	90.6	92.5	90.1
Allowed region (%)	9.4	7.1	9.1
Generously allowed (%)	0.0	0.0	0.8
Disallowed (%)	0	0	0
PDB accession code	5CBP	5B74	5B5U

Data-collection statistics for the crystals of cPfA at 37 °C, with peptide and dPfA with peptide.

Values in parentheses are for the highest resolution shell. The wavelength of the X-ray beam used for data collection was 1.5418 Å.

The interaction analysis showed that 2 salt bridges, 8 H-bonds and 86 hydrophobic interactions were present between these domains (Supplementary Table S2; Supplementary Fig. S2). Residues R175 and D178, reversibly formed a salt bridge while G134, T168, E177, L179, V181 and N182 were involved in hydrogen bond formation (Fig. 3B). Importantly, as compared to PfA, cPfA had double the number of inter-chain H-bonds and hydrophobic interactions, implying that the N-terminal domains of cPfA were more strongly associated with each other (Supplementary Table S2; Supplementary Fig. S2). However, it is likely that this specific association is pivoted around terminal β-strands in both cases (Fig. 3). The absence of the inter domain-linker in cPfA could have aided the “dissociated domains” to assemble into higher order structures with ease.

These results indicated that the site of association was similar in PfA and cPfA, the latter was more prone to form stable higher order protein assemblies with increase in temperature (Fig. 3B,C; Supplementary Table S2; Supplementary Fig. S2). This strong association might be possible in PfA but at a relatively higher temperature (> 70 °C). One of the probable reasons could be that the unstructured linker became more flexible above 70 °C facilitating a closer association of the N-terminal domains as shown by SAXS data (Fig. 2A). It is important to mention here that these N-N′ domain interactions between protein molecules are quite far from the active-site of the functional dimers as shown in Supplementary Figure S3.

### PfA undergoes β-strand mediated oligomerization and stabilization

To further investigate if the terminal β-strand of the N-terminal domain is important in providing stability through oligomerization under high temperature conditions, we truncated cPfA beyond D178 and named it as defunct cPfA (dcPfA) (Supplementary Fig. S1). We attempted crystallization of this protein between 18 and 37 °C in parallel with temperature based SAXS experiments. Our crystallization attempts of dcPfA were unsuccessful. Meanwhile, SAXS experiments provided the explanation for this failure. The scattering intensity profiles of SAXS data collected for dcPfA in the temperature range 25–40 °C showed negligible change in intensity, suggested that the shape of scattering particle was largely conserved (Supplementary Fig. S4A), however, the dimensions estimated through Guinier analysis showed that the size of protein dcPfA increased from ~ 5.2 nm (25 °C) to ~ 6.9 nm (40 °C) (Supplementary Fig. S4B). This increase in size was further enhanced at 50 °C where it was estimated to be ~ 12.1 nm. This expansion in size as well as the steep slope of the intensity profile at low Q region suggested the presence of scattering species of high molecular mass or aggregates (Supplementary Fig. S4A). It is important to mention here that at temperature > 50 °C, visible white aggregates were found in the sealed capillary, restricting further data acquisition at higher temperatures. As mentioned earlier, another observation made during this experiment was that the dimensions of dcPfA obtained at 25°dcPfA (~ 5.2 nm) were higher than those estimated for cPfA (R_g_ = 2.5 nm) studied under similar conditions. Probable explanation for the increased dimension could be: (1) the leftover ionic and hydrophobic interactions between N-terminal domains of dcPfA persisted even after removal of the last four residues of this domain; and/or (2) the N-terminal domain itself was capable of forming oligomers even at ambient temperature (around 25 °C), as reported earlier by our group^23^. Most probably, an increase in temperature triggered the formation of assemblies with non-specific oligomerization sites leading to decrease in stability of the protein rather than specific sites as present in PfA and cPfA which maintained their stability even at higher temperatures.

This concluded that in the absence of the short β-strand residues L^179^-V-V-N^182^ of the N-terminal domain, the dcPfA started losing its stability above 50 °C. In order to further validate this observation, we synthesized L-V-V-N peptide corresponding to the sequence of terminal β-strand and added in “trans” to dcPfA solution, to see its effect on the structure and functioning of the truncated protein.

### Crystallization of defunct cPfA (dcPfA) with peptide

**Figure 4:**
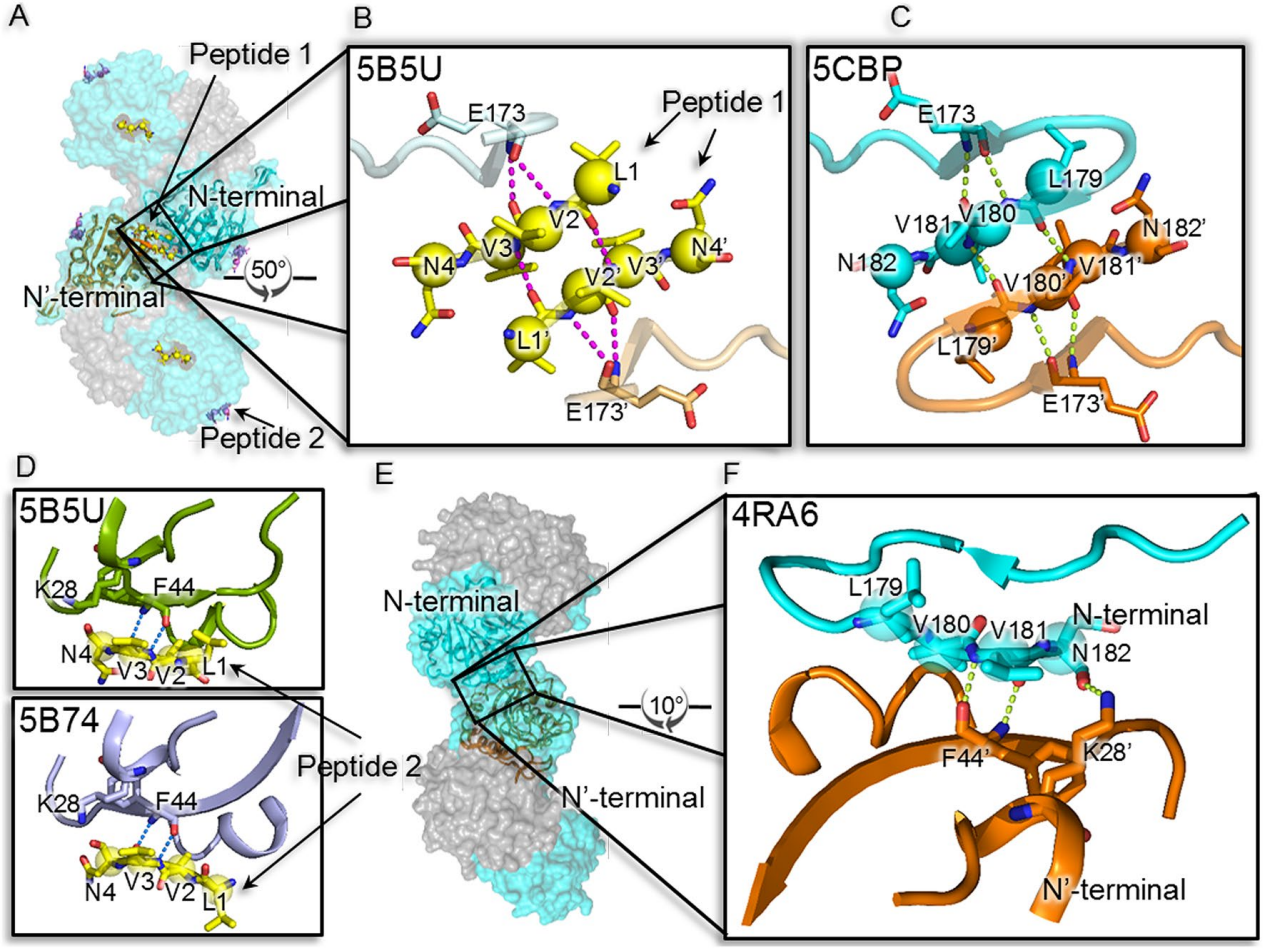
Effect of peptide on maintaining structural integrity of defunct PfA (dcPfA). (**A**) Surface representation (N-terminal domains in cyan and C-terminal domains in grey) of conjoined PfA (PDB ID: 5CBP) symmetry based tetramer superimposed on peptide- bound defunct PfA crystal structure (PDB ID: 5B5U). Interacting N-terminal domains of adjacent protein molecules are shown in cartoon representation (**B**), (**C**) marked as N (cyan) and N′ (orange) terminal domains. (**B**) Rotated and zoomed in-view of interaction interface showing intra and inter chain interactions between LVVN peptides and with their respective N-terminal domains in defunct PfA (PDB ID: 5B5U). (**C**) Similar view showing H-bonding interactions between adjacent LVVN sequences of conjoined PfA (PDB ID: 5CBP). (**D**) Zoomed in-view of the residues involved in H-bonding between N-terminal domain and peptide ‘2’ in defunct PfA with peptide (PDB ID: 5B5U, upper panel) and conjoined PfA with peptide bound structure (PDB ID: 5B74, lower panel). (**E**) Surface representation of ligand-less conjoined PfA (PDB ID: 4RA6)^19^ symmetry based tetramer superimposed on peptide-bound conjoined PfA (PDB ID: 5B74) and peptide-bound defunct PfA structure (PDB ID: 5B5U). (**F**) Zoomed in-view of the residues involved in H-bonding interactions between adjacent N-terminal domains of ligand-less conjoined PfA (PDB ID: 4RA6).

Since the dcPfA evaded crystallization, we attempted its co-crystallization with peptide L-V-V-N. Surprisingly, the crystals of this complex appeared under exact conditions where the cPfA crystallized previously (PDB ID: 5CBP). Diffraction data was collected up to 2.61 Å resolution (Table 2). wo additional electron densities were observed in both *f*_*o*_* − f*_*c*_ and *2f*_*o*_* − f*_*c*_ maps (Supplementary Fig. S5A,B). One of the electron densities was present at the same site where residues were truncated from cPfA (named e’ density 1) while the second was present near the residues 42–45 (named e′ density 2) (Supplementary Fig. S5). Post-refinement, LVVN peptides designated as P1 and P2 fitted well into the electron densities, 1 and 2, respectively with B-factor comparable to the interacting residues of the protein chain (PDB ID: 5B5U) (Fig. 4A; Supplementary Fig. S5A,B). The missing electron-density beyond R175 residue suggested that the last three residues (176–178) of N-terminal dcPfA became flexible in the absence of terminal β-strand (PDB ID: 5B5U) (Fig. 4B). Both cPfA (PDB ID: 5CBP) and dcPfA structures were identical with R.M.S.D. of 0.24 Å at Cα. Moreover, the peptide P1 was aligned to the corresponding sequence in cPfA with the R.M.S.D of 0.84 Å at Cα (Fig. 4B,C).

To delineate the interactions between P1 and dcPfA, the refined structure was submitted to EBI PDBsum server^22^ (Supplementary Fig. S6A). The results showed residue E173 of dcPfA was interacting with V2 of peptide (corresponding protein residue is V180) through two H-bonds (Fig. 4B). A similar interaction was present between E173 and V180 in cPfA (PDB ID: 5CBP) (Fig. 4C). Furthermore, P1 interacted through two H-bonds with the symmetry related peptide 1′ between L1 and V3′ and vice versa (PDB ID: 5B5U) (Fig. 4B; Supplementary Fig. S6). In cPfA, the corresponding residues i.e. L179 and V181′ were H-bonded (Fig. 4C; Supplementary Fig. S2). Thus, as all the interactions between P1 and dcPfA were identical to those present in cPfA, the peptide successfully restored the structural features of parent protein. Furthermore, peptide 2 (P2) was found to be interacting with N-terminal domain too where V2 and V3 residues of P2 were bound to N-terminal F44 through two H-bonds (PDB ID: 5B5U) (Fig. 4D, upper panel, Supplementary Figs. S4A,B, S6B). The interactions of P2 with dcPfA forced us to speculate that either this might be non-specific binding or probably another site involved in establishing contact between protein molecules. We thus attempted co-crystallization of cPfA with peptide to further investigate this observation.

### Substrate-induced oligomerization switch

We successfully co-crystallized cPfA and peptide and the refined structure proved that the P2 retained its binding site thus the interaction was specific. In this structure, the P2 interacted with the same residues, mainly F44 as seen in dcPfA (PDB ID: 5B74) (Fig. 4D, lower panel; Supplementary Figs. S5C, S6C). This observation suggested that the peptide binding at this site might also play a role in oligomerization of this protein. Considering these results, we again analyzed all the crystal structures of PfA and cPfA, across interacting symmetry mates^19^. Surprisingly, we found that in the crystal structure of cPfA where ligand was absent in the active site (PDB ID: 4RA6)^19^, the symmetry based N-terminal domains interacted through three H-bonds between V181, N182, F44′ and K28′ residues along with 48 non-bonded contacts (Fig. 4E,F; Supplementary Table S2; Supplementary Fig. S2). All these interactions were located in a single zone between adjacent protein molecules. This result solved the riddle regarding binding of peptide (P2) at the same site in both peptide-bound dcPfA and cPfA structures. It also suggested that this region was the site of interaction between protein molecules if the active site remain unoccupied by any substrate/ligand. As reported previously, the unstructured active-site loop in ligand-free cPfA (PDB ID: 4RA6) became structured once the active site was occupied by the ligand in PfA (PDB ID: 4Q0M) and cPfA (PDB ID: 4NJE and 4RA9)^19^. The structured active sites of adjoining protein molecules established contact through H-bonds and hydrophobic interactions (Supplementary Fig. S7A,B). This caused a conformational change in the functional dimer due to the loss of interactions of V181 and N182 with F44′ and K28′, respectively, in substrate-free conditions (Fig. 4F). However, the residues 178–182 formed a newer and stronger interactions with similar residues of adjacent protein molecules as described in the beginning for PfA and cPfA (Fig. 3A–C). Thus, in the presence of substrate, protein molecules were involved in interaction at two sites: the active-site loops and the tail region of N-terminal domains. In contrast, in the absence of substrate, only one site of interaction was possible. Also, the number of interactions between protein molecules were approximately double indicating that they were capable of forming more stable assemblies in the presence of substrate (Supplementary Table S2). In conclusion, though, this protein existed in an oligomeric state under high temperature conditions, it is more likely that conformational changes in the presence of substrate significantly enhanced its tendency to form stronger interactions and higher order associations.

### Functional-loss in dcPfA and peptide-mediated activity restoration

**Figure 5:**
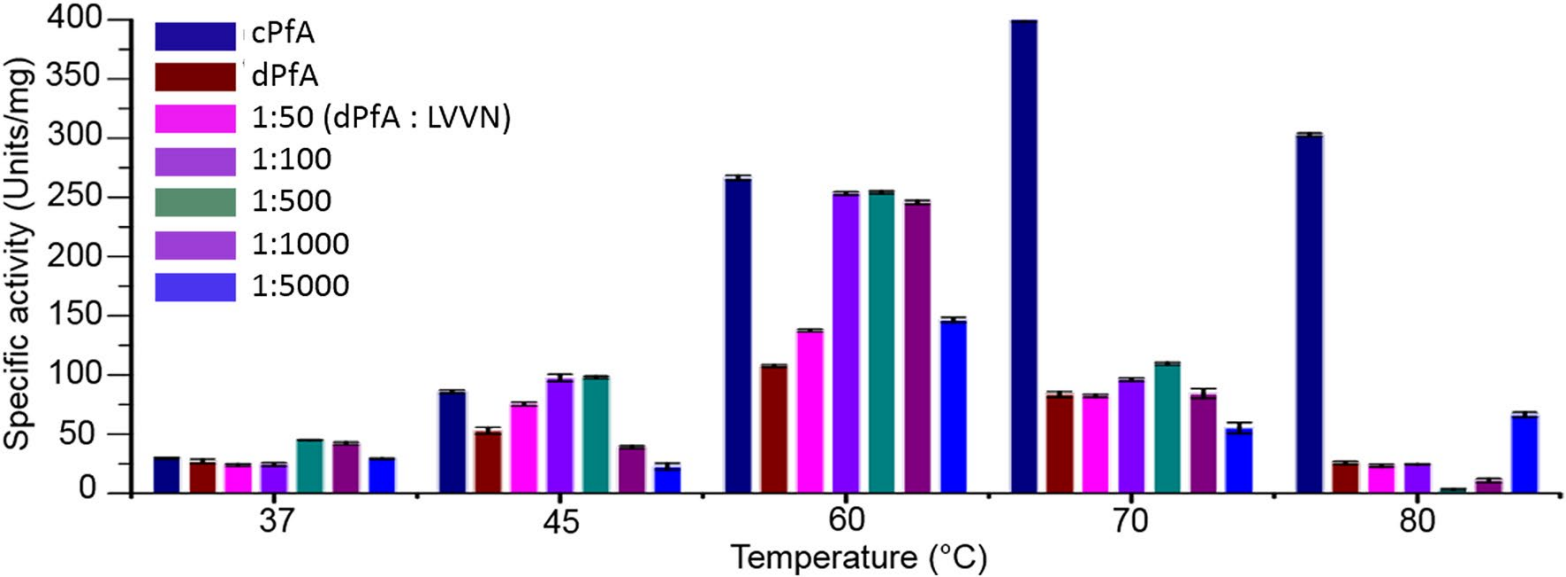
Effect of peptide (LVVN sequence; corresponding to terminal short β strand of N-terminal domain of PfA) in restoring the activity of defunct PfA (dcPfA) under different temperature conditions. In the absence and presence of different molar ratio of peptide, the specific activity of defunct PfA (dcPfA) is compared with conjoined PfA (cPfA).

We further investigated the effect of heat treatment and N-terminal sequence deletions on the specific activity of dcPfA. Until 60 °C, dcPfA showed a somewhat proportional rise in activity with temperature (i.e*.* twofold as compared to threefold increase in cPfA), beyond which the activity started decreasing. Importantly, prominent differences were discerned in terms of approximately 38%, 60%, 80% and 90% loss in specific activity in dcPfA, relative to cPfA, at temperatures 45 °C, 60 °C, 70 °C and 80 °C, respectively (Fig. 5). Maximum loss in specific activity was seen at higher temperatures, most likely due to break down of important H-bonded associative interactions between the N-terminal domains of separate functional dimer assemblies (Fig. 3). As a consequence, the overall stability of native dimers also got hampered, which was in accordance with the SAXS data findings. To validate further the role of the terminal short β-stretch in sustaining the higher order native-associations, the synthetic peptide L-V-V-N was supplemented in dcPfA assay reactions. Surprisingly, the reaction samples where peptide in different molar ratios was pre-incubated with dcPfA in the temperature conditions, 37–60 °C (as depicted in Fig. 5), the specific activity was radically high. At the highest effective peptide concentration i.e. 500 µM, the specific activity increased by 1.66-fold, 1.85-fold and 2.36-fold at 37 °C, 45 °C and 60 °C, respectively, as compared to dcPfA alone (Fig. 5). Such peptide-mediated activity restoration depicted that the peptide probably binds the same sites where these residues were intact originally in cPfA (supported from dcPfA-peptide bound crystal structure data, Fig. 4B; Supplementary Figs. S5, S6), thereby reconciling interactions between native functional assemblies. At 37 °C and 45 °C, the dcPfA in the presence of peptide showed specific activity higher than cPfA (about 1.5 fold increase at 500 µM peptide concentration, respectively), probably due to the presence of an additional oligomerization site as observed in the dcPfA-peptide bound crystal structure (PDB ID: 5B5U) (Fig. 4D upper panel; Supplementary Figs. S5B, S6A). At higher temperatures (70 °C and 80 °C), it is possible that peptide could not locate the binding site in protein because of high kinetic energy and as a result, the specific activity did not increase further, except a marginal increment at higher peptide concentrations. Importantly, analogs LAAN and LVAA were not able to restore the activity in dcPfA, further supporting that the interactions between peptide LVVN and the protein were specific (Supplementary Fig. S8). Likewise, in the case of PfA and cPfA also at 60 °C, a significant increase in specific activity was observed in the presence of peptide which is again possibly due to enhanced molecular associations via peptide binding at an additional site (PDB ID: 5B74) (Fig. 4D lower panel; Supplementary Figs. S5C, S6C, S9).

## Discussion

**Figure 6:**
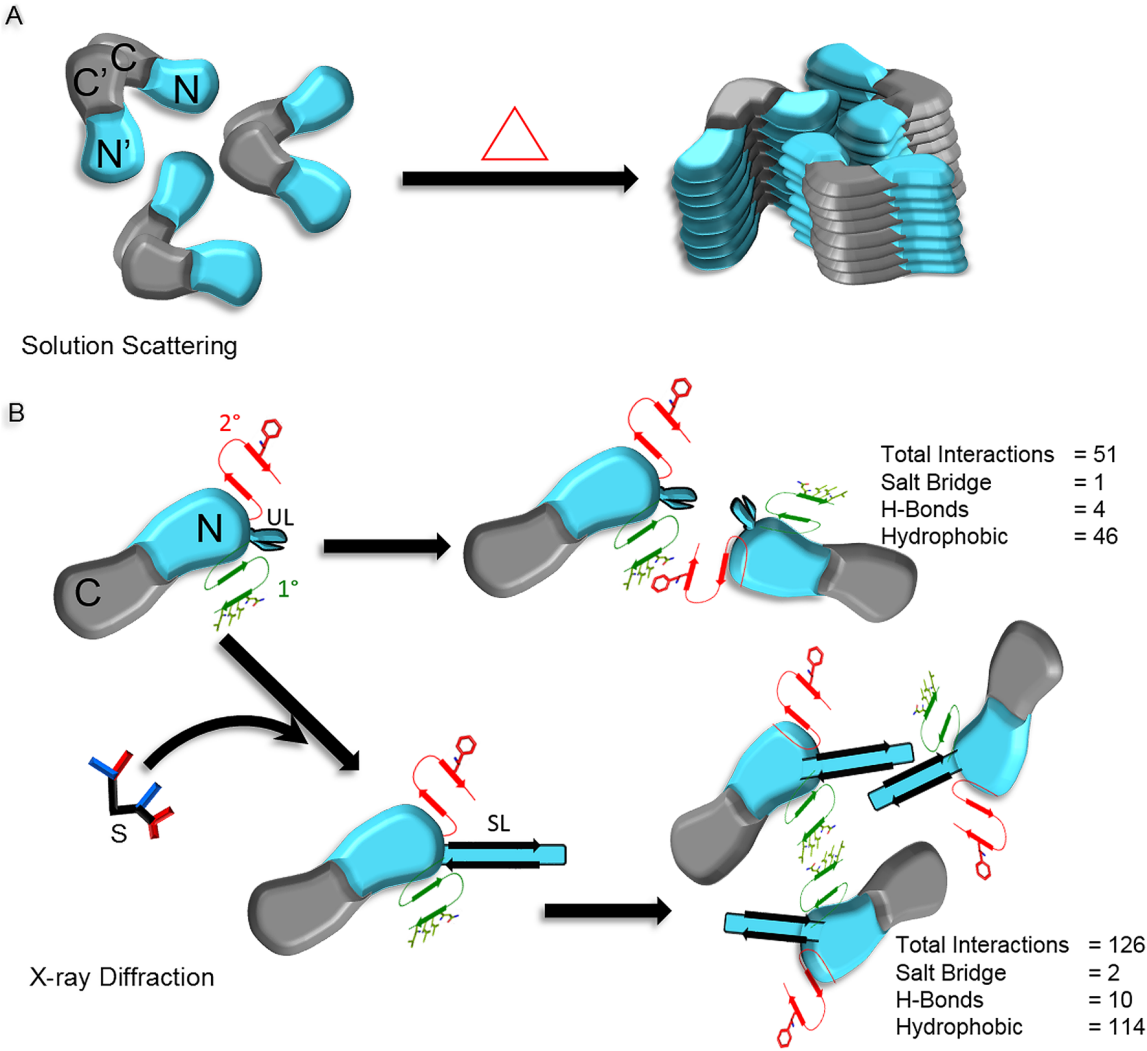
Schematic representation of mechanism of l-asparaginase oligomerization. (**A**) Solution scattering data shows that protein molecules undergo oligomerization with heating. (**B**) X-ray diffraction data helped in depicting the probable sites and mechanism of intermolecular associations deduced from the crystal structures of PfA variants in the substrate-free and bound conformations. 1° and 2° denotes primary and secondary sites involved in intermolecular associations. UL and SL denotes unstructured and structured active site loop. S denotes substrate.

To understand the structural basis of the molecular mechanism adopted by hyperthermophilic PfA for thermoprotection, we first performed solution scattering experiments between 25 and 80 °C. An increase in scattering intensity observed above 60 °C showed that the size of scattering species increases upon heating. The oligomerization of the protein is considered the most suitable explanation for the observed increase in dimensions given that this protein is highly stable up to 80 °C, thus ruling out the possibility of unfolding^24^. Such quaternary protein structure arrangement is thought to be an important adaptation acquired by thermophilic organisms during evolution to protect from heating (Fig. 6)^9,11,25–27^. Failed crystallization attempts at higher temperatures (> 37 °C) prompted us to analyze the previously reported low temperature crystal structure of this protein to get insights into the most probable sites where protein molecules can interact during oligomerization. The symmetry based crystal structure analysis showed that the adjacent dimers were interacting through N-terminal domains, a characteristic well supported by an earlier report which proved that the N-terminal domain of PfA is capable of oligomerization in isolation^23^. The terminal residues of this domain which form a β-strand were prominently involved in the oligomeric interaction. Importantly, the dimensions and the structure of the symmetry-based tetramer matched well with the models of scattering species.

The linker-less variant i.e. cPfA with intact terminal residues of N-terminal domain, was found to be equally stable and even more active under high temperature conditions than the wild type PfA as reported earlier^19^. Therefore, to get the structural insights into the oligomeric forms, we attempted crystallization of the cPfA at higher temperature. Symmetry analysis of the refined cPfA structure at 37 °C (PDB ID: 5CBP) revealed that the adjacent functional assemblies were interacting through N-terminal domain residues and the interactions were twice than the wild type protein PfA. This structure was identical to the crystal structure of cPfA at 18 °C (PDB ID: 4RA9)^19^ with R.M.S.D. at Cα ~ 0.11 Å. Thus, an increase in temperature had negligible effect on its structure. Furthermore, the symmetry-based N-terminal domains were bound by similar interactions as present at 18 °C. The absence of linker in this protein might have provided more flexibility in the alignment of N- and C-domains within the functional dimer assembly and as a result, the interactions between the N-N′ domains of different functional dimers became much stronger. The solution scattering experiments supported this assumption where scattering molecules with dimensions similar to the tetramer were found above 50 °C in the case of cPfA but in the case of PfA were observed above 70 °C. Further rise in temperature led to the formation of higher order oligomeric states in cPfA which can be attributed to the stability provided by strongly bound adjacent N-terminal domains. These results also suggested that, in PfA, the heating may lead to increase in linker flexibility which may pave way for the direct interaction between terminal β-strands of N-terminal domains leading to the formation of stable tetrameric species ≥ 70 °C (Fig. 6). Thus, the formation of much higher order oligomers and the absence of linker seems to be the most probable reasons for the previously observed higher activity of cPfA relative to PfA, where oligomerization was limited up to tetramers even at 80 °C^19^.

Symmetry analysis of the previously reported PfA and cPfA structures revealed that the terminal β-strand of the N-terminal domain is probably involved in providing stability to the higher order oligomeric states in these proteins. Therefore, to rule out the possibility of crystallization artifacts and confirm that the terminal β-strand is involved in oligomerization, we made a defunct version of cPfA (dcPfA) that was devoid of the last four residues i.e. LVVN, which spans the terminal β-strand of the N-terminal domain. Crystallization attempts of dcPfA were unsuccessful, however, solution scattering results as a function of temperature showed that the protein aggregated above 50 °C (Supplementary Fig. S4). Lack of specific oligomerization sites probably led to random associations and this might have increased entropy of the system decreasing the stability of dcPfA significantly. Later on, successful crystallization of dcPfA with LVVN peptide where latter retains its location at the speculated site (where the terminal β-strand was originally present in the parent protein) re-established the structural stability and thus also restored functionality. In conclusion, the short terminal β-strands stitch together higher order native associations in PfA variants (Fig. 4).

The presence of an additional peptide binding site in cPfA or dcPfA and the symmetry-based interaction analysis of available crystal structures led us to propose the two discrete mechanisms of the higher order assembly formation depending upon the absence and presence of the ligand (as shown in schematic): (1) in the absence of substrate, the primary (1°; β-strand tail) and secondary (2°; F44 residue position) sites are involved in interaction with each other (corresponding to P1 and P2 binding site, respectively) leading to the formation of oligomers and (2) in the presence of substrate, the conformational changes occur at the active site loop which switch the interaction interface. Upon binding to the substrate, the protein association takes place through 1°–1°′ (tail-to-tail) and the active site loop-to-loop′ interactions leading to the formation of stronger and higher order oligomers (as shown in schematic). Such rearrangement would help protein molecules to stabilize under high temperature conditions.

These results thus confirm that the terminal β-strands of N-terminal domain play a crucial role in providing the stability to PfA/cPfA by establishing higher order associations at elevated temperatures. In conclusion, the findings reported here provide structural evidence in support of temperature induced higher order oligomer formation conferring stability to thermophilic proteins thereby regulating their functioning. These higher order oligomers may act as energy barriers restricting the protein from aggregation. However, the enhanced activity observed in all the PfA variants in the presence of peptide needs further investigation. Nevertheless, the findings of this study may pave way to rationally engineer proteins and help design small molecules that can enhance stability thereby may modulate activity of the proteins of commercial importance.

### Experimental procedures

#### Expression and purification of PfA, cPfA and dcPfA

Cloning, expression and purification of PfA and cPfA was performed according to optimized protocol mentioned in earlier reports^19,20,24^. For obtaining dcPfA, the last four residues starting from 179 to 182 from the protein sequence of N-PfA were removed by introducing a stop codon just after the 178th residue in the reverse primer DNA sequence so that after PCR, DNA sequence of desired length could be amplified. Subsequently, cloning of DNA sequences corresponding to both deleted NPfA and CPfA domains was done using the protocol mentioned earlier^19^. Positive clones of both domains, after confirming through DNA sequencing, were separately transformed in *E. coli* Rosetta (DE3) and expression of recombinant proteins was checked on SDS-PAGE. A standard Ni–NTA-affinity based purification procedure under denaturing conditions (Qiagen protocol) was followed and subsequently, co-refolding of both the domains was done as described previously^19^. The protein samples were purified through gel filtration chromatography using Superdex S-200 column (GE Healthcare) attached to an AKTA purifier FPLC system (GE Healthcare). Protein concentration was determined by UV absorbance at 280 nm (A_280_, _1__mg/mL_ ~ 0.72) for all the proteins, PfA, cPfA and dcPfA.

### SAXS data collection and analysis

All SAXS data presented in this work on PfA, cPfA and dcPfA have been collected at SAXSpace system (Anton Paar GmbH, Graz, Austria) with line collimated source and data was recorded on a 1D Mythen detector (Dectris, Switzerland). For data collection, protein samples at concentration of 6 mg/mL and their matched buffers were loaded in lockable thermostated quartz capillary. Approximately, 50 μL of sample was loaded and using a TCS stage, variable temperature SAXS experiments were done. Protein samples and matched buffer were heated stepwise from 25 to 80 °C. Three exposures of 15 min were averaged for each dataset. For datasets, beam position correction was done using SAXStreat program. Subsequent data processing including buffer subtraction and desmearing was done using SAXSquant software as described previously^28^. Data analysis was done using PRIMUSQT software package in ATSAS 2.7.1 which included estimation of different shape parameters like radius of gyration (R_g_), maximum linear dimension (D_max_), histogram of various interatomic vectors, and intensity values at zero angles (I_0_)^29^. Shape reconstruction was done using integrated plugin for DAMMIF, DAMAVER and DAMMIN programs^30^. For shape restoration, ten models of uniform density were calculated using SAXS I(Q) profile as reference, superimposed, aligned and averaged using DAMAVER and the final solution was re-optimized using DAMMIN program. Superimposition of SAXS data based models with crystal structure and models generated using symmetry mates were done using SASPy plugin for PyMOL program^31^. All figures for this work were prepared using PyMOL program^22,25,29^.

### Crystallization

All proteins were purified and concentrated to 10 mg/mL for crystallization set-ups. PfA and dcPfA did not crystallize under high temperature conditions. Crystallization setups of cPfA were done as reported earlier^19^. Post-setup, crystallization plates were transferred to vibration free RUMED unit maintained at 37 °C, 45 °C and 50 °C temperature. Screening of plates was done on alternate days to check for crystal growth. Crystals appeared at 37 °C after 2 days of setting up, were left to grow in size, for additional 10 days. Above 37 °C, cPfA failed to yield any diffractable crystals. Co-crystallization of dcPfA and cPfA with LVVN peptide was carried out under conditions where crystals of cPfA were obtained previously^19^. Both, dcPfA and cPfA proteins (6 mg/mL) were mixed with peptide in 1:1, 1:10, 1:100 and 1:1000 molar ratios, separately. After set-up, the small crystals appeared after 16 days in the wells having 1:10 and 1:100 protein to peptide ratio. These crystals were allowed to grow for another 15 days. Diffraction data was collected from crystals obtained from 1:10 protein to peptide ratio.

### X-ray diffraction data collection

Diffraction data of cPfA crystals grown at 37 °C was collected on RIGAKU MicroMax-007HF^19^. Initially, crystals were mounted in a capillary at room temperature for diffraction data acquisition. Initial data analysis show that the unit cell parameters of these crystals were similar to cPfA crystals grown at 18 °C (PDB ID: 4RA9) reported previously^19^. Exposure to X-rays at room temperature induced damage in crystals, so upon conformation that cell parameters were similar to previous cPfA crystals, diffraction data was collected under stream of liquid nitrogen maintaining temperature close to 100 K. Crystals were soaked in cryo-protectant solution containing 15% glycerol added to the corresponding mother liquid just before diffraction. Sample to detector distance was 200 mm, 200 and 175 mm for crystals of cPfA grown at 37 °C, dcPfA+ peptide and cPfA+ peptide, respectively. Each frame was collected for 10 min with 1° oscillation. Diffraction data processing including intensity integration and scaling was done using HKL2000 and iMosflm programs^32,33^.

### Structure refinement

Using PDB 4RA9^19^ as a search model, estimation of the number of chains in the asymmetric unit and the space group was determined using Matthews Coefficient and PHASAR programs of CCP4i suite, respectively^34–36^. Initial structure determination was done by molecular replacement method using MOLREP program (CCP4i suite)^37^. The models were initially refined by rigid body refinement followed by restrained refinement using REFMAC5^36^. Further refinement and model building was done by using COOT^38^ and PHENIX^39^ programs until complete models were achieved. Solvent molecules present in the solution were added once R_w_ value reached around 0.25. Molecules were added to electron densities where F_o_ − F_c_ map had more than 3σ value above the mean, forming at least one hydrogen bond with protein or other solvent atom. PROCHECK was used for validation of the refined models^40^.

### Peptide synthesis

LVVN and its analogs, LAAN and LVAA were synthesized using standard solid phase peptide synthesis employing Fmoc (N-(9-fluronyl)-methoxycarbonyl) chemistry in 0.02 mol scale on a Protein Technologies Inc., USA, PS-3 peptide synthesizer^41^. The chain elongation of the peptides was done by using four molar excess of the protected Fmoc-amino acid with HBTU (2-(1H-Benzotriazole-1-yl)-1,1,3,3-tetramethyluronium hexafluorophosphate) as a coupling reagent and HOBT (n-hydroxybenzotriazole) or COMU (1-Cyano-2-ethoxy-2-oxoethylidenaminooxy) dimethylamino-morpholino-carbenium hexafluoro-phosphate) to suppress racemization^42,43^. For C-terminal activation, 0.4 M NMM in DMF was used and for N-terminal Fmoc-group deprotection 20% piperidine in DMF along with 0.5% HOBT was used. After final Fmoc-group removal the peptides were cleaved from the resin by treating the resin with a cleavage cocktails containing Trifluroacetic acid (TFA) and scavengers. Finally, they were purified to homogeneity using Dionex Ultimate 3000 HPLC connected with a reverse phase C-18 semi-prep and analytical column using water/acetonitrile gradient containing 0.1% TFA and then lyophilized to obtain about 9–11 mg of purified peptides. Intact molecular masses of the peptides were confirmed by Voyager MALDI-TOF and were found to be in close agreement with their theoretical ones (data not shown).

### Activity assay

Enzymatic activity was measured for the wild type (PfA), conjoined (cPfA) and defunct PfA (dcPfA) using standard Nesslerization protocol as used earlier^44^. Briefly, 6.25 µl of protein sample from stock concentration of 4.2 µM (to yield final protein concentration, 0.0525 µM in a reaction mixture of 500 µl) was pre-heated with different molar concentrations of peptide solution for one hour at different temperatures (37 °C, 45 °C, 60 °C, 70 °C and 80 °C) for 60 min. A control reaction, devoid of peptide, was set up for all the proteins under identical conditons. Separately, a reaction mix containing 50 µl of substrate (stock concentration 0.1 M) and 50 mM sodium phosphate pH 7.4 was also pre-equilibrated for 5 min at different temperatures. Thereafter, to this reaction mix, protein-peptide solution (or protein alone) was added and incubated for further 10 min at the same temperature. Enzymatic reaction was stopped by adding 25 µl of 10% TCA. For remaining steps, a previously reported protocol was followed and finally, the activity was measured in terms of units/mg^19^.

## Supplementary Information

Supplementary Information
