# Supplementary material for: Heat induces end to end repetitive association in P. furiosusl-asparaginase which enables its thermophilic property
Source: Sci Rep. 2020 Dec 10;10:21702. doi: 10.1038/s41598-020-78877-z (PMC7728782; doi:10.1038/s41598-020-78877-z)
Supplement: Supplementary file 1 — Supplementary Information [file 41598_2020_78877_MOESM1_ESM.pdf]

SUPPORTING INFORMATION

HEAT-INDUCES END-TO-END REPETITIVE ASSOCIATION IN *P. furiosus* L-ASPARAGINASE WHICH ENABLES ITS THERMOPHILIC PROPERTY

PANKAJ SHARMA[1§], RACHANA TOMAR[2§], SHIVPRATAP SINGH YADAV[1], MAULIK D. BADMALIA[1], SAMIR KUMAR NATH[1], ASHISH*[1] AND BISHWAJIT KUNDU*[2]

[1]CSIR–INSTITUTE OF MICROBIAL TECHNOLOGY, CHANDIGARH AND [2]KUSUMA SCHOOL OF BIOLOGICAL SCIENCES, INDIAN INSTITUTE OF TECHNOLOGY DELHI INDIA

[§] These authors contributed equally

Table S1: SAXS data collection and scattering parameters

**Data-collection parameters**	
Instrument	SAXSpace (Anton Paar)
Beam geometry	10 mm slit
Wavelength (Å)	1.5418
Desmearing Software	Done using SAXSquant
q range (nm^{-1})	0.10–3.00
Temperature (K)	298-353
**Data analysis and modelling programs employed**	
Beam Position Correction	SAXStreat
Primary data reduction	SAXSquant
Data processing	PRIMUS QT
Ab initio analysis	DAMMIF
Validation and averaging	DAMAVER
Computation of model intensities	CRYSOL
Three-dimensional graphics representations	PyMOL

Table S2: Interactions between N-terminal domains of symmetry related PfA structures

Crystal structure	No. of salt bridges	No. of H-Bonds	No. of Hydrophobic interactions
**PfA with ligand (4Q0M)**	2	4	44
**cPfA with ligand (18°C)(4RA9)**	0	10	83
**cPfA with ligand (37°C)(5CBP)**	2	8	86
**cPfA without ligand (4RA6)**	1	3	48
**Interactions at active site loop region**			
**PfA with ligand (4Q0M)**	0	2	18
**cPfA with ligand (37°C)(5CBP)**	0	2	28

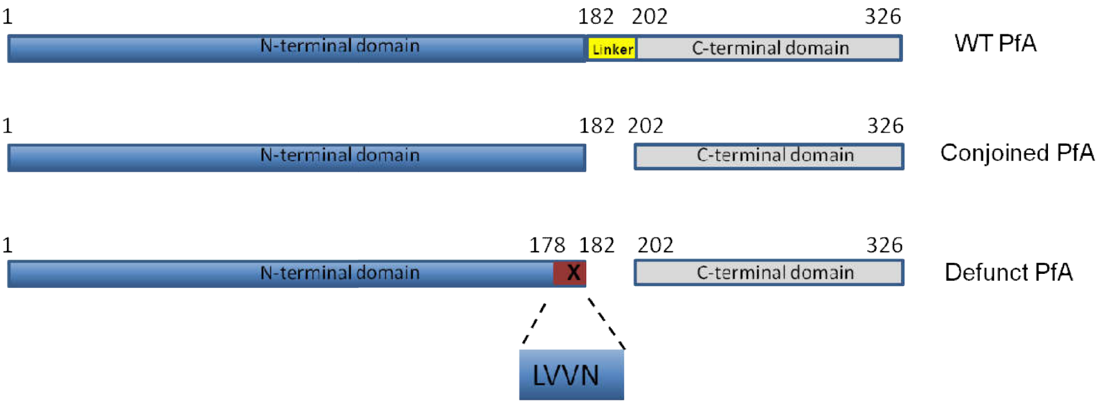

Figure S1 Cartoon figure showing domain architecture of PfA and its variants.

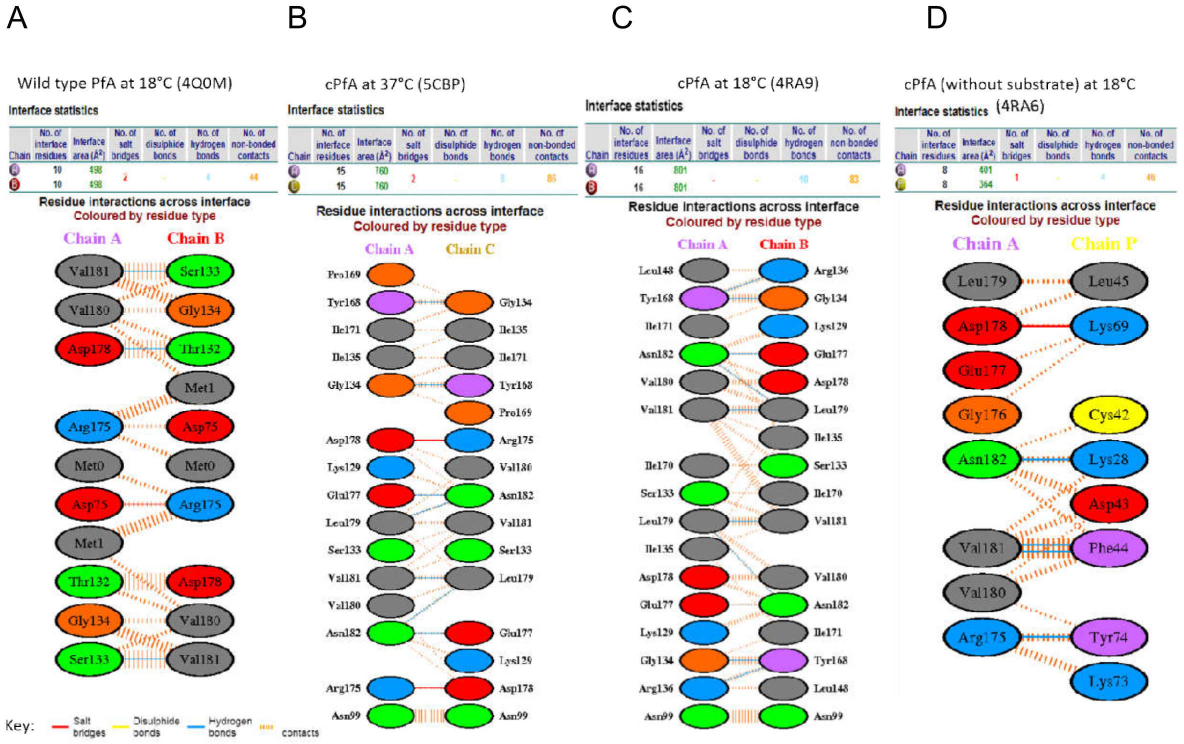

Figure S2. PDBsum (18) results: Symmetry based N-N' terminal interactions in PfA variants at different temperatures. **(A)** Interactions in wild type PfA, crystals were grown at 18°C, (PDB: 4Q0M). **(B)** Interactions in cPfA, crystals were grown at 37°C (PDB: 5CBP) **(C)** Interactions in cPfA, crystals were grown at 18°C (PDB: 4RA9)**. (D)** Interactions in cPfA (without substrate/ligand at active site), crystals were grown at 18°C (PDB: 4RA6). It is worth mentioning here that except **(D)**, all the structures have either substrate or ligand bound to the active site of protein. Since, all the chains represent N-terminal domains, chain names are different in the figure with respect to crystal structures to ensure clarity.

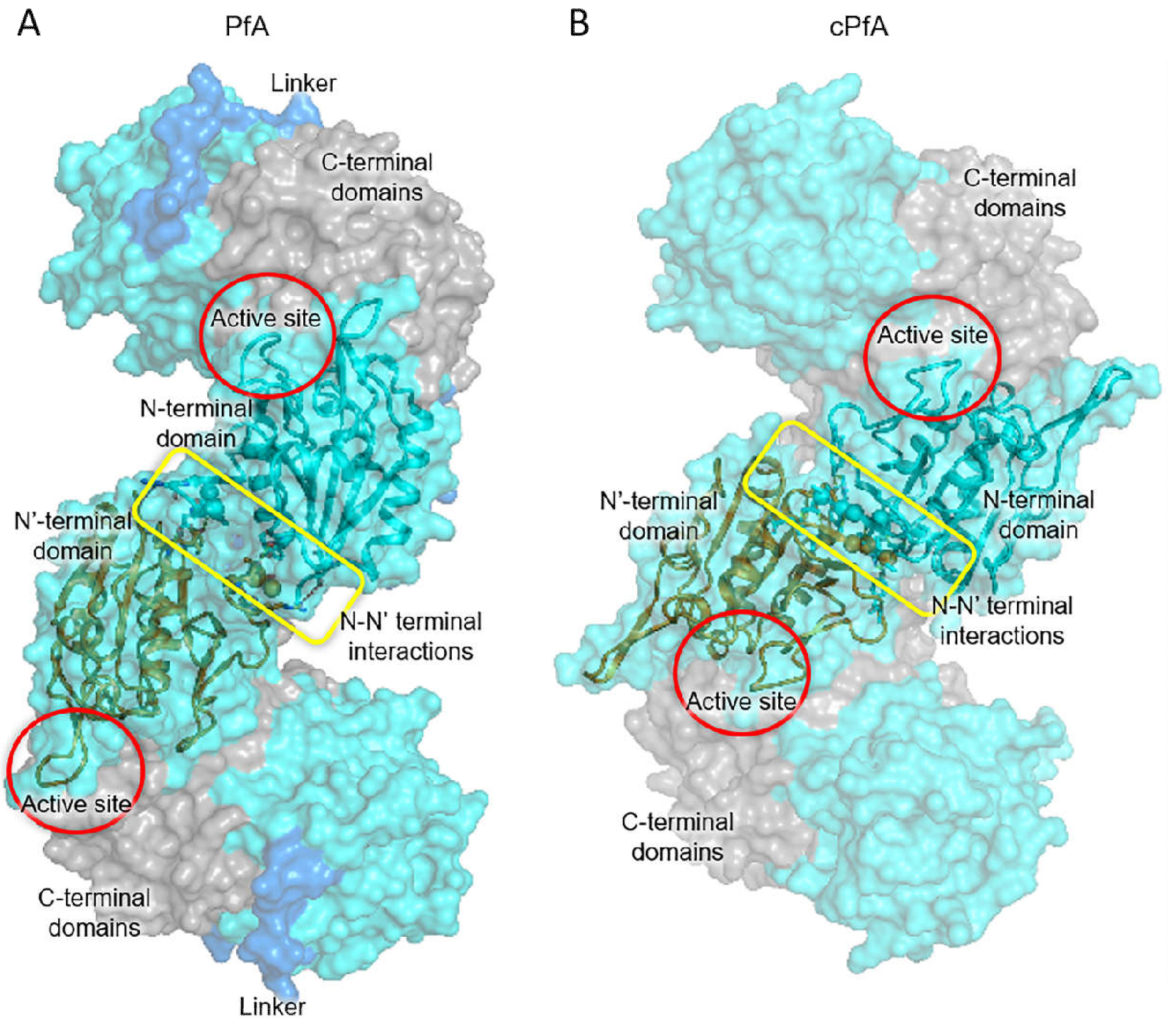

Figure S3 Location of oligomerization and active sites on PfA and cPfA tetramers. **(A)** The active site of PfA is shown in red circle and the N-N' terminal oligomerization site is shown in yellow rectangle. **(B)** Similarly, the active site and the N-N' terminal oligomerization of cPfA is shown in red circle and yellow rectangle, respectively.

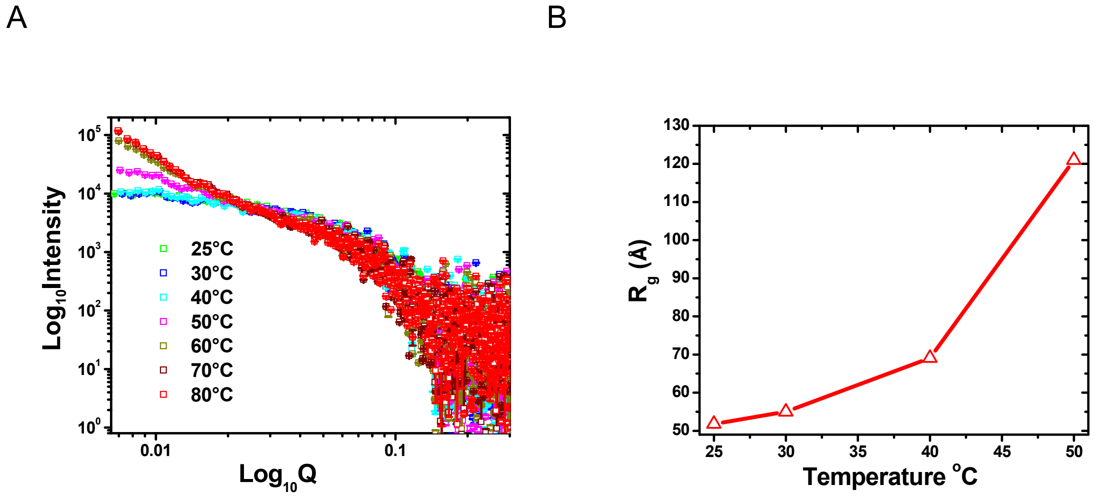

Figure S4. Solution scattering data of defunct cPfA (dcPfA) as a function of temperature **(A)** The SAXS intensity data acquired for 6mg/ml protein between 25 to 80°C. Steep increase in intensity above 50°C shows that protein aggregated above this temperature. **(B)** Effect of temperature on dimensions *i.e.* R_G of protein. Dimensional analysis was not done above 50°C as protein undergoes aggregation.

A B C

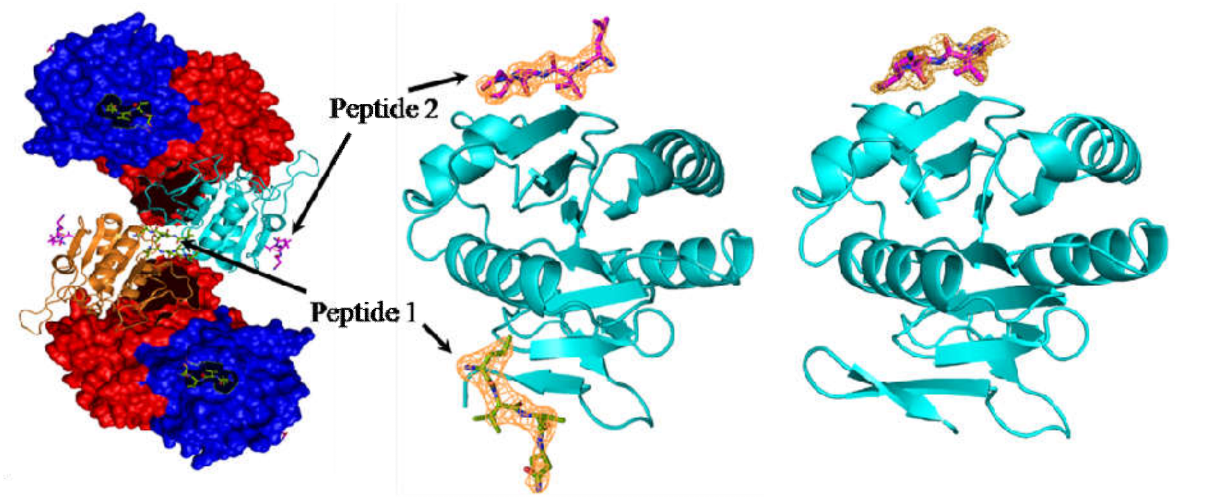

Figure S5 Effect of peptide in restoring structure integrity of dcPfA. **(A)** Symmetry based tetramer of crystal structure of dcPfA (PDB: 5B5U) with two bound peptides at N-terminal domains. **(B)** N-terminal domain with bound peptide molecules, peptide 1 (e' density 1) and peptide 2 (e' density 2) (PDB: 5B5U). **(C)** N-terminal domain of cPfA with bound peptide molecule in similar orientation (PDB: 5B74). $2F_o - F_c$ map of electron density at 1 σ level is shown in orange mesh for peptides in both (B) and (C).

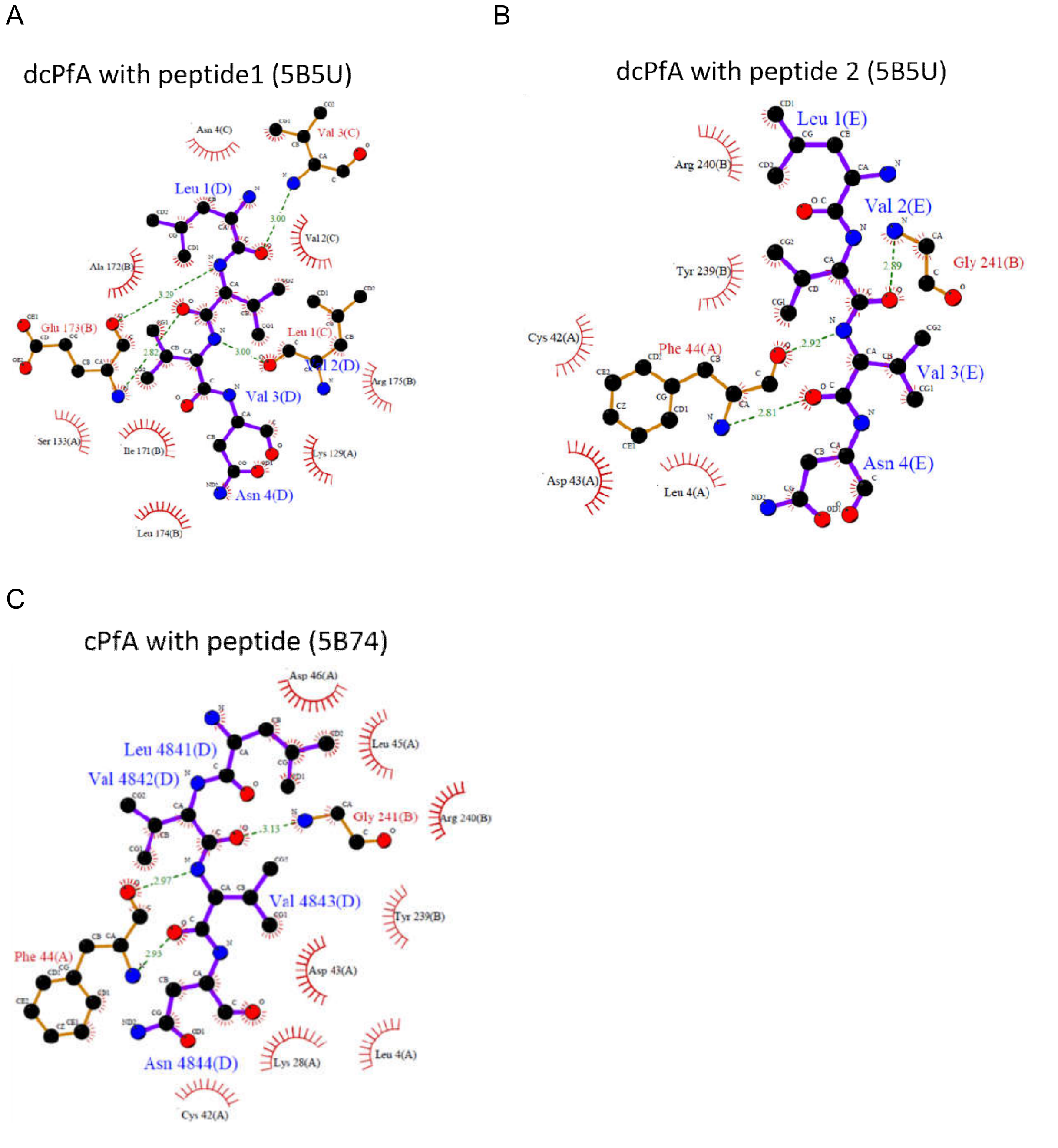

Figure S6. PDBsum (18) based LIGPLOTS of peptide interactions. **(A)** Residues involved in interaction with peptide1 in dcPfA (PDB: 5B5U). **(B)** Residues involved in interaction with peptide2 in dcPfA (PDB: 5B5U). **(C)** Residues involved in interaction with peptide in cPfA (PDB: 5B74).

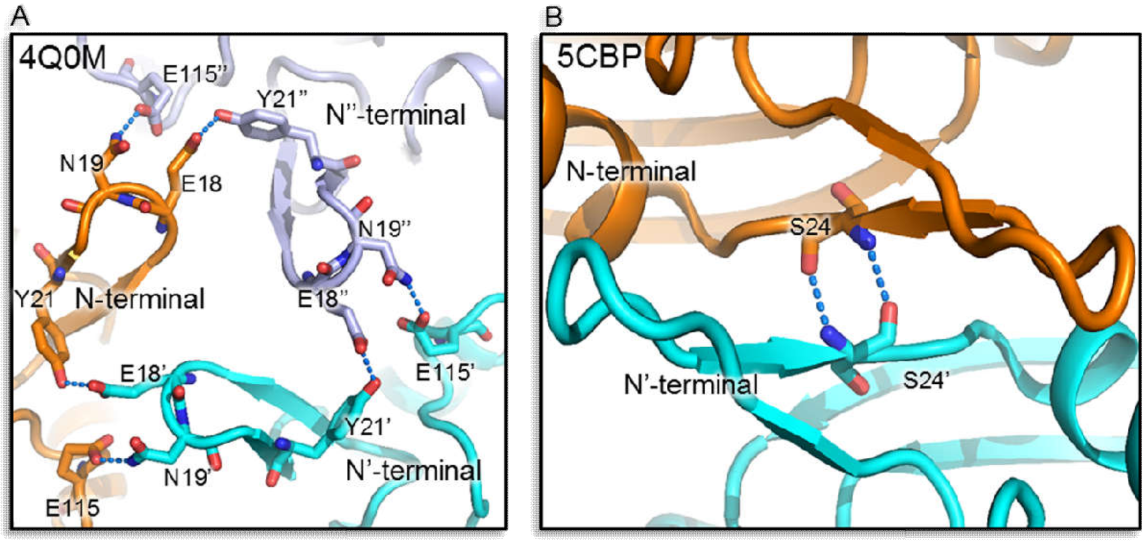

Figure S7 Active site loop-loop interactions between symmetry related molecules of different PfA variants. Ligand induced loop-loop interactions at **(A)** structured active site of symmetry based trimer of PfA (PDB ID: 4Q0M) and **(B)** symmetry based dimer of cPfA (PDB ID: 5CBP).

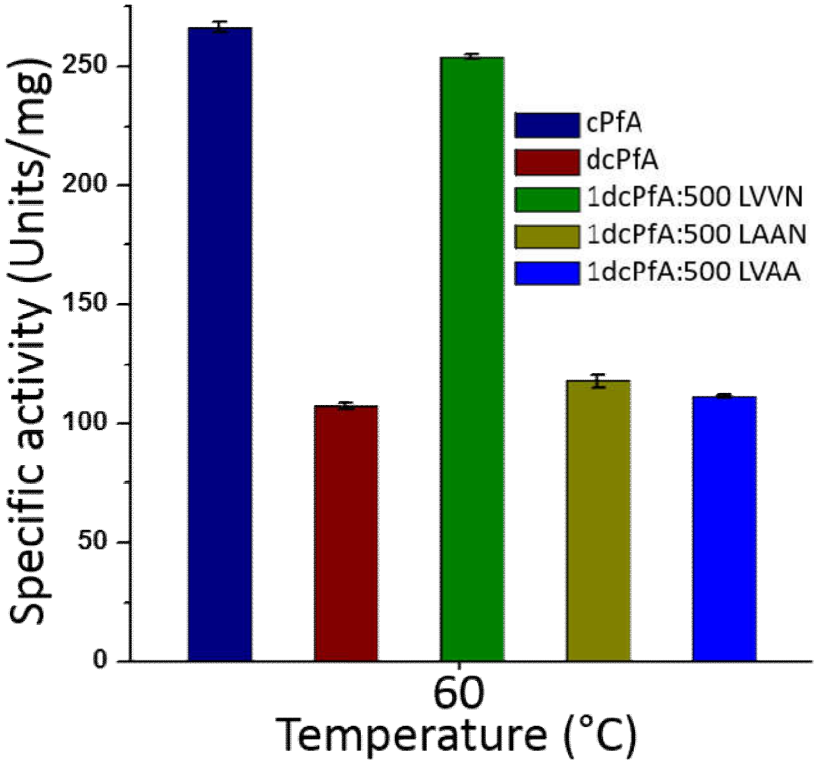

Figure S8 Effect of peptides LVVN, LAAN and LVAA on the activity of dcPfA at 60°C. At 500 molar concentrations, both analog peptides did not enhance the activity of dcPfA.

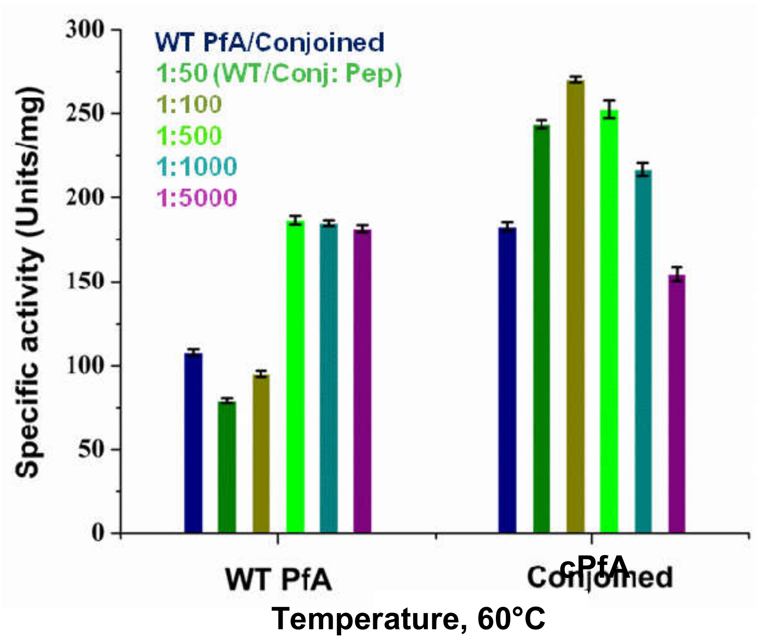

Figure S9 Effect of peptide on the activity of wild type PfA and conjoined PfA (cPfA) at 60°C. At certain molar concentrations, peptide enhances the activity of both the proteins.
